# Validation of biomarkers of diabetic retinopathy for preventing and predictive medicine in diabetic complications

**DOI:** 10.1186/1878-5085-5-S1-A79

**Published:** 2014-02-11

**Authors:** Folami Lamoke, Sean Shaw, Babak Baban, Anna Lisa Montemari, Francesco Facchiano, Livia Di Renzo, Giovanni Parisi, Guido Ripandelli, Mario Stirpe, Manuela Bartoli

**Affiliations:** 1 Department of Pharmacology and Toxicology –Georgia Regents University- Augusta, GA 30912 –USA; 2 Department of Ophthalmology – Georgia Regents University- Augusta, GA 30912- USA; 3 Department of Oral Biology – Georgia Regents University- Augusta, GA 30912- USA; 4 Department of Experimental Medicine and Pathology – University of Rome “La Sapienza” – Rome, Italy; 5 Department of Hematology and Oncology - Istituto Superiore di Sanita’ - Rome, Italy; 6 IRCCS Fondazione GB Bietti- Rome, Italy

## Scientific object

The epidemic diffusion of diabetes mellitus has raised serious concerns about the deleterious effects of its complications. Diabetic retinopathy (DR) is a severe progressive ocular pathology and the leading cause of blindness in adults. There is a significant limitation in specific biomarkers for DR, thus, implying that DR diagnosis occur when vision is already compromised. Poor glycemic control, with elevated hemoglobin A1c (HbA1c), and hypertension are recognized risk factors, however the implication of inflammatory processes in DR pathogenesis has prompt interest in the identification of biomarkers of disease within the immune system.

## Technical approach/methods

By using flow cytometry and multiplex ELISA assays, we have analyzed a large number of inflammatory cytokines and determinants of innate immunity in serum of diabetic patients at different stages of DR. In addition, we have analyzed and compared the expression pattern of these factors in post-mortem retinas and vitreous of diabetic donors.

## Results/interpretation

A quite large number of pro-inflammatory factors were up-regulated in the serum of diabetic patients, but only a restricted number of them were correlated with clinically overt DR. We have focused our attention on three factors and pursued more studies on their contribution to DR induction and progression. The results of these studies have determined that toll-like receptor 4 (TLR4), interleukin 17 (IL-17) and uricemia are all potentially involved in promoting auto-inflammatory activity both systemically and in retina of diabetic patients. In particular, while elevation of TLR4 expression and activity along with increased uric acid levels were predictive of DR induction in all stages, increased levels of IL-17 was found in patients affected by most severe stages of DR such as macular edema and proliferative diabetic retinopathy. These data support the hypothesis that monitoring of pro-inflammatory factors in serum of diabetic patients held predictive value for DR. In addition, our results, suggest that a definite set of factors are directly associated with different stages of retinopathy in diabetic patients.

